# Epidemiological assessment of Respiratory Syncytial Virus infection in hospitalized infants, during the season 2005–2006 in Palermo, Italy

**DOI:** 10.1186/1824-7288-35-11

**Published:** 2009-05-02

**Authors:** Paola Di Carlo, Amelia Romano, Ludovico Salsa, Alessandra Gueli, Antonella Poma, Fortunata Fucà, Piera Dones, Mirella Collura, Diego Pampinella, Delia Motisi, Giovanni Corsello

**Affiliations:** 1 Dipartimento di Scienze per la Promozione della Salute, Università degli Studi di Palermo, Palermo, Italy; 2 Malattie Infettive, Ospedale dei Bambini "G. Di Cristina", ARNAS Civico, Palermo, Italy; 3 Medicina d'Urgenza, Ospedale dei Bambini "G. Di Cristina", ARNAS Civico, Palermo, Italy; 4 Fibrosi Cistica, Ospedale dei Bambini "G. Di Cristina", ARNAS Civico, Palermo, Italy; 5 Dipartimento Materno Infantile, Università di Palermo, Palermo, Italy

## Abstract

**Objectives:**

Respiratory Syncytial Virus (RSV) is the leading cause of hospitalization for lower respiratory tract infections (LRTI) in young children worldwide.

We evaluate the epidemiological and clinical patterns of RSV infection in infants hospitalized for LRTI in in Palermo, South Italy, Sicily.

**Methods:**

We collected the demographic details of infants hospitalized to G. Di Cristina Children's Hospital in Palermo for LRTI between November 2005 and May 2006. We also included all cases occurred in newborns hospitalized in the Neonatal Intensive Care Unit (NICU) Of Palermo.

**Results:**

During the studied period, 335/705 hospitalized infants for LRTI were enrolled in the study. The trend of hospitalization started in late winter and lasting until May 2006 with an epidemic peak in spring. 178/335 infants tested for viral infection showed RSV disease. Three cases occurred in preterm newborns hospitalized from birth in NICU. The likelihood to be RSV+, rather than RSV negative (RSV-) was higher for infants < 6 months and lower for infants with history of breast feeding (P < 0.05). RSV infection was associated with a higher likelihood to be admitted to intensive care unit and to a longer hospitalization and oxygen therapy.

**Conclusion:**

The study shows that, in Sicily, RSV is an important cause of LRTI in infants. The seasonal distribution shows that both LRTI and RSV infections peak in late spring, in contrast to Northern Italy. Our data could help to define the regional appropriate start of prophylactic interventions.

## Introduction

Almost all children are infected by Respiratory Syncytial Virus (RSV) at least once by 2 years of age, and approximately 1–2% of infants will require hospitalization for RSV-associated Lower Respiratory Tract Infections (LRTI) [1-3].

Infection rates vary from 50–70% during the first year of life, to 100% during the second-third year of age. Immunity, however, is not complete, and reinfection is common [1-4].

Evidence of RSV infection has been found in every geographic area studied. In countries with a temperate climate, outbreaks generally occur during the autumn and last until spring, with the number of infections peaking in January-March. In tropical areas, epidemics generally coincide with the rainy season [5-7].

Although palivizumab can reduce the rate of hospitalization due to RSV-LRTI, it is costly to administer, therefore local data need to be evaluated to formulate guidelines and justify its use, especially in countries with economic difficulties [8].

In Italy, RSV outbreaks begin in the late winter and last until spring, reaching a peak in March.

Recent climate changes have influenced the regional trend of the country, altering the start and peak of the RSV epidemic season [9,10].

### Objective

the Authors conducted this study to evaluate the epidemiological and clinical patterns of RSV infection in infants hospitalized for LRTI in in Palermo, South Italy, Sicily.

## Methods

### Patients

A prospective surveillance study was performed from October 1, 2005 to April 30, 2006. The study population consisted of children admitted to the "G. Di Cristina", Children Hospital of Palermo.

Consent for enrolment was sought from parents by study personnel. All children less than 2 years of age who were hospitalized with symptoms suggesting LRTI were enrolled in the study. LRTI were categorized on the basis of clinical and roentgenographic findings, according to the criteria proposed by Ruuskanen and Ogra [11]. The disease was diagnosed: a) as wheezy bronchitis, when an acute illness characterized by cough, rhonchi, and expiratory wheezing was detected; b) as bronchiolitis, when wheezing dyspnea, tachypnea and CXR hyperinflation of the lung with or without areas of collapse were present; c) as pneumonia when radiographic findings of lung parenchymal involvement, with interstitial-alveolar infiltrates and/or consolidation, were found. The radiologist and the clinicians allocating patients to these categories were blinded as to RSV status.

Demographical, clinical and microbiological data were prospectively collected and entered into an Access Database (Microsoft). The following parameters were recorded: demographic characteristics, breast-feeding, history of underlying diseases, history of admissions for respiratory problems, start and duration of symptoms, need for intensive care, oxygen supplementation, results of microbiology tests and chest radiograph.

We also evaluated the incidence of LRTI in all newborns hospitalized in the N.I.C.U. of the 'Dipartimento Materno Infantile-IMI' and the 'V. Cervello' Hospital in Palermo during the same period. Informed consent was obtained from a parent or guardian. The study was approved by the Ethics Committee of the ARNAS Hospital of Palermo, Italy.

### Oximetry

Oxygen saturation (SaO2) and pulse rate were determined upon admission and then daily, using a portable Nellcor oximeter (Nellcor, Inc., Hayward, CA). Data were always collected for at least 5 min (except for longer periods of observation when measurements were not stable or not considered reproducible), with the child awake and quiet in room air and, when needed, in oxygen. Oxygen was administered when SaO2 was <90% and amounts of oxygen supplementation were recorded.

Admission in Intensive Care Unit (ICU) was decided when the cardio-respiratory or the general conditions of the patients required intensive care.

### Virologic Studies

Nasopharyngeal swabs and throat specimens were collected at the time of LRTI and tested for a panel of respiratory viruses by immunofluorescent test (PathoDx^® ^Respiratory Virus Panel Kit, Immunofluorescent test for 7 respiratory viruses: influenza A; influenza B; adenovirus; parainfluenza 1, 2, 3; and RSV) in shell vial pre-CPE assays, and conventional tube culture confirmation (CE, Remel, Lenexa, KS 66215. USA), or for RSV alone by enzymatic diagnostic test (Now^® ^RSV Test, Binax) according to medical request-record.

### Statistical analysis

Analysis was based on comparison of demographic data between RSV + and RSV - infants. Between groups comparison was performed by means of chi-square test for categorical variables and by Student's *t*-test for continuous variables collected in the study.

Data were processed with a SPSS-pc statistical program. A significance level of p < 0.05 was used throughout the study.

## Results

**Figure 1:**
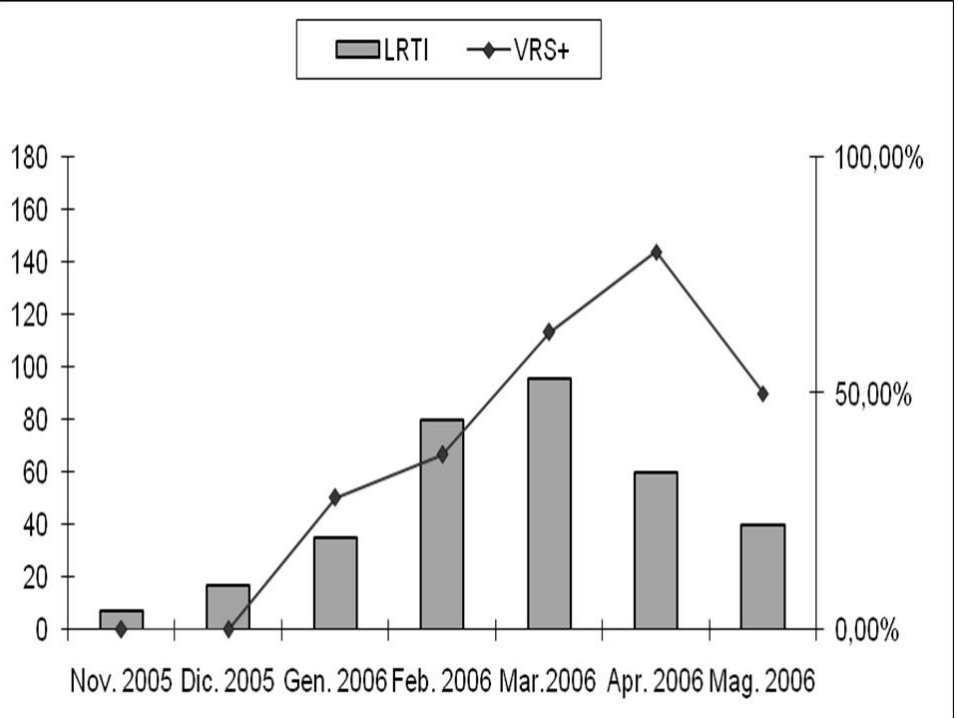
**Seasonal trend of RSV infection in 335 enrolled infants**.

Of the 1219 children referred to the Emergency Unit of the 'G. Di Cristina' Children's Hospital between November 2005 and May 2006, 705 (58%) were hospitalized with suspected LRTI. The trend of LRTI hospitalization started in November 2005, lasting until May 2006 with an epidemic peak in March (figure 1). Of these 705 children, n°35 were over 2 years old and n°25 had a history of two or more previous hospitalizations and were carried out from the study.

Of the remaining 645 infants, only 335 showed viral investigation and were enrolled in the study.

The retrospective interview of the pediatricians on because the viral investigation did not perform in the excluded patients has showed that in 60% of the cases the patients have been hospitalized in the weekend when viral analysis was not routinely available, in 10% of the cases the infants were early tested, in 3% of the cases the parents have refused the investigation and in the remaining 27% of cases the clinical, instrumental and epidemiological investigations were considered suitable for the management of LRTI. The percentage of exclusion showed an equally distribution with the trend of hospitalization.

RSV was identified in 178/335 patients (53%), parainfluenza type 3 virus in 14, influenza A virus in 10 and adenovirus in 2 cases. No combined virus infection was observed. The epidemic peak of the LRTI occurred in March, with a prevalence of RSV positivity of 63% raising up to 80% in April, while no RSV positivity was seen in October, November or December (figure 1).

The characteristic of 335 studied infants are shown in table 1.

**Table 1:** Demographic and clinical data of the 335 RSV studied infants in the season of November 2005–April 2006

	RSV Positive (n = 178)	RSV Negative (n = 157)	P
%Male (n = 214)	59	41	> 0.05
%Female (n = 121)	73	17	> 0.05
Age (months)	3 ± 1.4	5 ± 7.1	**<0.05**
%Age at time of hospitalization < 3 months (n = 132)	63	37	> 0.05
%Age at time of hospitalization < 6 months (n = 220)	59	41	**<0.05**
%Age at time of hospitalization > 6 months (n = 115)	43	57	> 0.05
Gestational age (weeks)	38 ± 1,7	38 ± 1,9	> 0.05
%Gestational age >36 weeks (n = 315)	45	55	> 0.05
Birth weight (gr.)	2986 ± 680	3023 ± 609	> 0.05
Birth weight group > 2500 g (n = 291)	44.3	55.7	> 0.05
Previous Hospitalization for LRTI (n = 97)	37	63	> 0.05
Breastfeeding (n = 187)	58	129	**<0.05**
Days of hospitalization	6.4 ± 3.5	5.6 ± 3.1	**<0.05**
Days of oxygen therapy (n = 235)	4.9 ± 3.3	4.1 ± 2.3	**<0.05**

Data are shown as percentage or means ± SD. The value of p refers to statistical significance of differences between the two populations;

Independently from the RSV positivity, when evaluating the age distribution, 132 (39%) of them were less than 3 months old and 220 (65%) were less than 6 months old.

Four (1%) infants were born before 32 weeks gestation, 16 (5%) were born between 33–35 weeks and the remaining 315 patients (94%) were born after the 36th week. All infants born before 32 weeks gestation were RSV-negative and received palivizumab prophylaxis. None of the RSV positive children received it. The distribution of gestational age (GA) showed that in our study population it was <36 weeks in 6% of the subjects versus 4.0% in general population [12,13].

Evaluating the distribution of birth weights, 4 infants weighed under 1.500 g, 10 between 1.500–2.000 g, and 30 between 2000 and 2.500 g and the remaining 291 infants over 2.500 at birth.

187 of the 335 children (55,8%) were breast-fed for more than 3 months.

Of the 335 enrolled children, 224 (67%) had diagnosis of bronchiolitis, 35 (10%) had pneumonia, and 76 had wheezy bronchitis (23%); respectively, 55% of the patients with bronchiolitis, 16% of those with pneumonia, and 29% of those with wheezy bronchitis showed RSV infection.

One infant was affected by bronchopulmonary dysplasia and 4 were affected by congenital cardiopathies.

During the studied period, three cases of RSV was identified in 3 preterm newborns (2 patients born at 28 weeks gestation and 1 born in the 34th week) hospitalized from birth in the neonatal intensive care unit (NICU). The RSV respiratory diseases occurred before discharge when they stayed in post-intensive area.

The age at time of admission was lower in RSV + rather than in RSV-negative (RSV.) infants (p = 0.00). The likelihood to be RSV+, rather than RSV-negative (RSV-), was higher for infants < 6 months (p = 0.00) and lower in infants with a history of breast-feeding (p = 0.02) (table 1). Other demographic and clinical characteristics were evaluated but were not significantly different between RSV+ and RSV- infants. Comparing severity of disease, a tendency toward longer hospitalization and a longer oxygen therapy were detected in RSV+ infants when compared with RSV-infants. (Table 1).

Furthermore, 10 RSV + infants were admitted to the Pediatric Intensive Care Unit, on average for 11.3 days versus no one infant in the RSV - patients. Only 1 patient required assisted ventilation.

## Discussion

Previous Italian surveillance study of acute respiratory infections (ARI) in hospitalized infants had showed that respiratory syncytial virus is the prevalent etiological infectious agent [10]. The incidence of ARI tracked closely with the RSV season, with the majority of infections occurring from late winter through early spring.

RSV is a significant cause of morbidity leading to the hospitalization of children in Palermo, particularly infants under 2 years of age. The seasonal distribution of our patients shows that RSV infections peak in late spring, in contrast to Northern Italy. This pattern is similar to that reported for tropical countries, where RSV infection also occurs in the summer [5,14].

Efforts to prevent this infection are based on case management, vaccination and the identification of risk factors.

Furthermore, in our report, we attempted to delineate clinical pattern of RSV + hospitalized infants in a population of south Italian children who experience extremely high rates of hospitalization for LRTI. Our results show that age at time of admission for LRTI was lower for infants RSV + rather than RSV- (3 months versus 5 months) and the age 0–6 months increased the risk of RSV hospitalization.

This is not surprising as a chronological age of 3 months or less at the onset of the RSV season is a known risk factor for severe RSV-induced LRTI [12-14]. In addition to the small size of the conducting airways and incomplete development of the lung structure, these infants are less likely to have RSV-neutralizing maternal antibodies than infants born after the peak of the RSV season [15]. Lung growth and a more efficient immune response may explain why a chronological age of > 6 months at the time of hospitalization is associated with a lower likelihood of being hospitalized for a LRTI due to RSV [14-16].

In our report, the study of LRTI population revalues the protective effects of breast-feeding to RSV infection, recently underlined by some studies.

It is not clear how breast-feeding reduces the risk of infection, although immunomodulatory constituents of human milk seem to be protective [17].

Although recent immunological studies have tried to define the mucosal and/or systemic mechanisms of protection against respiratory infections during the early months of life, more studies are required to identify which elements modify the evolution of disease. Therefore the protective role of breast-feeding must be carefully considered [17,18].

The statistical significant association between RSV infection and days of hospitalization and oxygen therapy confirms that this infection is cause of severity illness and that prompt recognition of the diagnosis that include early treatment with oxygen therapy helped to improve the clinical state of the children [10,19-21]. In particular, hospital stay greater than 5 days is considered one of relevant data for score range of illness severity [21,22].

In accordance with this observation is the remark that in our study all infants hospitalized in ICU were RSV+.

Nosocomial RSV infection were observed in NICU during the studied season. These findings showed the relevant role of viral infection to determine severe respiratory illness in hospitalized patients. Several studies conducted in both community and healthcare settings have also demonstrated that promoting hand-washing and good hygiene practices to be adopted when coughing can help reduce the transmission of acute respiratory infection (ARI) [22]

Due to the low number of children with specific characteristics, other risk factors such as presence of chronic lung diseases or of congenital heart disease, congenital or acquired immunodeficiency, haematological malignancies, bone-marrow or organ transplants, and cystic fibrosis were not analyzed.

There were two major limitations in our study. Firstly, no clinical score was used in our hospital for children with LRTI when the children were admitted to the Paediatric Infectious Disease Unit and clinical data collected. Secondly, this study reports the results of only one tertiary care medical center in Palermo. A comprehensive study including local clinics, regional hospitals and medical centers would provide more details of RSV infections in Sicily.

In conclusion, RSV is the most important viral pathogen in infants and children under the age of 2 years. The seasonal trend of RSV infections in Palermo is "atypical", with peaks in the late winter and spring.

Furthermore, the data provided by the study on the expected onset and end of the RSV season indicates that palivizumab prophylaxis start should be discussed keeping in mind the substantial variability in community RSV season timing.

## Competing interests

The authors declare that they have no competing interests.

## Authors' contributions

PD conceived of the study, participated in its design and coordination and drafted the manuscript. AR conceived of the study and participated in its design and coordination. LS carried out the virologic studies. AG collected demographical, clinical and microbiological data of the children hospitalized in the Division of Infectious Diseases of the "G. Di Cristina" Children's Hospital of Palermo. AP collected demographical, clinical and microbiological data of the children hospitalized in the Division of Infectious Diseases of the "G. Di Cristina" Children's Hospital of Palermo. FF collected demographical, clinical and microbiological data of the children referred to Emergency Unit of the "G. Di Cristina" Children's Hospital of Palermo. PD participated in the coordination of the study. MC collected demographical, clinical and microbiological data of the children hospitalized in the Cystic Fibrosis Center of the "G. Di Cristina" Children Hospital of Palermo. DP performed the statistical analysis. DM collected demographical, clinical and microbiological data of the children referred to Emergency Unit of the "G. Di Cristina" Children's Hospital of Palermo. GC conceived of the study and participated in its design and coordination. He also collected demographical, clinical and microbiological data of the children hospitalized in the N.I.C.U. of the "Dipartimento Materno Infantile-IMI" and the "V. Cervello" Hospital of Palermo. All authors read and approved the final manuscript.
